# E-cigarette Vaping is Associated with Pro-Fibrotic Gene Expression in Kidney and Liver Tissues

**DOI:** 10.21203/rs.3.rs-7609713/v1

**Published:** 2025-10-06

**Authors:** Wanjun Gu, Howard Chang, Poorvi Saini, Samvel Gaboyan, Jarod Olay, Jorge A. Masso-Silva, John Shin, Ira Advani, Ashley Du, Cameron Brand, Joan Heller Brown, Laura E. Crotty Alexander

**Affiliations:** 1 Department of Neurology, Weill Institute for Neurosciences, University of California San Francisco, San Francisco, CA 94158, USA; 2 Pulmonary and Critical Care Section, Medicine Section, VA San Diego Healthcare System, La Jolla, CA 92161, USA; 3 Division of Pulmonary, Critical Care and Sleep Medicine and Physiology, Department of Medicine, University of California, San Diego, La Jolla, CA 92093, USA; 4 Department of Pharmacology, University of California, San Diego, La Jolla, CA 92093, USA

**Keywords:** Fibrosis, E-cigarette, Tobacco, Kidney, Liver, Transcriptomics

## Abstract

Conventional tobacco use causes a wealth of diseases and adversely affects cells and organ systems across the body. The long-term effects of e-cigarette vaping on the same remain unclear and identifying early pathogenic signals at the organ level via animal models may shed light on potential downstream effects in humans. Here we investigate transcriptomic changes in the kidney and liver, organs known to be damaged by long-term combustible tobacco use, of mice exposed daily to e-cigarette aerosols (vapor) with or without nicotine. C57BL/6 male 6–8 week-old mice underwent whole-body exposure to room air, e-cigarette (3^rd^ generation box mod) vapor containing 70:30 propylene glycol and glycerin (70:30 PG:Gly) without nicotine (Vehicle), and e-cigarette vapor with 6 mg/mL nicotine in 70:30 PG:Gly (E-cig) for 1 hour daily for 3 months. RNA sequencing on kidney and liver tissues and apriori gene set analysis were performed. Unbiased principal component analysis identified closer clustering of E-cig and Vehicle groups, relative to Air, in the kidney. Assessment of the a priori gene set found nicotine to be associated with greater transcriptomic changes in the kidney while vehicle chemicals induced greater changes in the liver. Alterations in expression of *Car3* and *Foxo3* identify oxidative stress and inflammation in the renal system caused by e-cigarette exposure, while dysregulated *Il6ra* and *Lpin1* in the liver highlight disruptions in lipid metabolism and immune signaling. Chronic inhalation of e-cigarette vapor alters gene expression in downstream organs, in a pattern most consistent with promotion of fibrosis and metabolic dysregulation, underscoring the need to define long-term pathophysiologic effects of e-cigarette vaping across the body.

## Introduction

**Figure 1.**
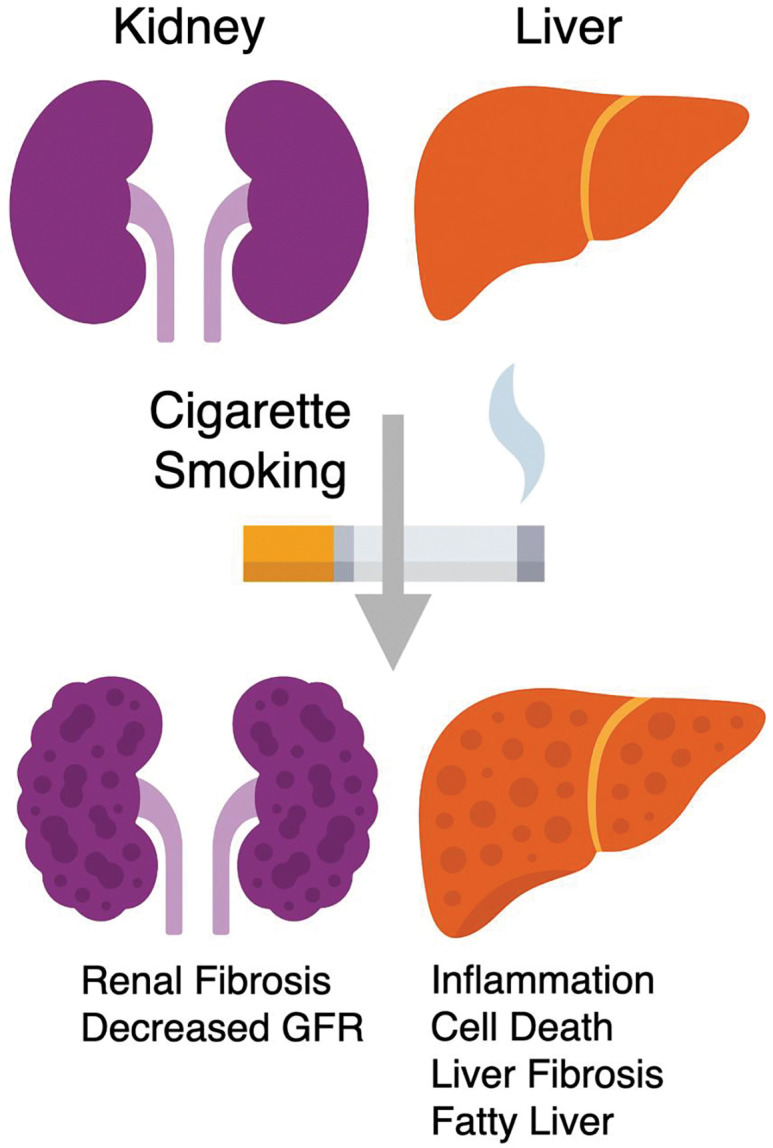
Chronic inhalation of tobacco smoke is known to adversely affect both the renal and hepatic organ systems, leading to fibrosis and diminished function.

Inhalation of combustible tobacco smoke is known to cause numerous adverse health effects, including hepatorenal toxicity^1–4^. For example, exposure to combustible tobacco smoke is associated with higher rates of renal failure^1,5,6^, renal fibrosis^3,7,8^, liver failure^9^, and liver fibrosis^10,11^. The pathway by which these organ-damaging effects occur is via tissue injury caused by the 4–7,000 toxic chemicals within tobacco smoke, followed by tissue repair complicated by fibrosis^4^ (Figure 1). While much is known about the molecular, cellular and tissue-level effects of combustible tobacco use, electronic (e)-cigarettes or vaping devices are a newer form of tobacco for which we are still determining the health effects.

Because e-cigarettes have only been in regular use since the mid 2010s, it will be many more years before we identify health effects of chronic vaping via epidemiologic methods. Murine models have yielded helpful data regarding the impact of daily e-cigarette aerosol (commonly called vapor) inhalation, including findings that e-cigarette vaping alters responses to respiratory pathogens^12–14^, alters cardiopulmonary physiology^15–17^, and leads to gene expression changes across the body^18–24^. Few studies have focused on the potentially renal toxic effects of e-cigarettes. However, the data thus far indicates that e-cigarette vaping does cause kidney damage and diminishes renal function^25^. In our previous studies, we found that “vape pen” (2^nd^ generation) e-cigarette aerosol exposures 1 hour daily for 3–6 months increased circulating proinflammatory and pro-fibrotic proteins, decreased renal filtration rates, and induced fibrosis in the kidneys and livers of female mice, both in C57BL/6 and CD-1 backgrounds^24^. Another study found that chronic exposure to nicotine containing e-cigarette vapor in the setting of a high-fat diet increased inflammatory responses, oxidative stress-induced DNA injury, and pro-fibrotic markers in mouse kidneys, suggesting accelerated development of renal pathology^26^. Interestingly, no-nicotine (Vehicle) also led to renal pathology, but via suppression of mitochondrial OXPHOS complexes and extracellular matrix deposition, which is likely to cause structural instability.

In terms of effects of e-cigarettes on the liver, one study utilizing a murine model found nicotine containing e-cigarette exposures to be associated with hepatic steatosis in adult offspring while non-nicotine e-cigarette exposures (Vehicle) led to metabolic changes and liver damage in dams and offspring^27^. An in vitro study, thus of lower physiologic relevance, of Kupffer cells exposed to e-cigarette extracts identified a robust inflammatory response, oxidative stress production and cytokine release^28^. Both studies used a similar generation of e-cigarette (3^rd^) and nicotine type (base nicotine) and concentration (6–18 mg/mL) as the current study. A study of e-cigarette flavorant chemicals found that vanillin, ethyl vanillin, and ethyl maltol caused cytotoxicity in HepG2 cells, concerning for possible hepatotoxicity in human vapers^29^. These data suggest that multiple e-cigarette chemicals cause liver inflammation, which is commonly a first step in pathogenesis of liver fibrosis, or cell death (which can also lead to inflammation). Also, these data show that nicotine containing e-cigarettes cause different pathology than Vehicle (no nicotine) e-cigarette exposures.

The majority of e-cigarettes and e-liquids on the market have not been thoroughly assessed for health effects, particularly for adverse effects outside of the lung. To define the impact of long-term, daily inhalation of e-cigarette aerosols on gene expression in kidneys and liver, as a precursor to pathologic effects on organ structure and function that we have previously identified, we conducted daily exposures to e-cigarette aerosols for 12 weeks. To continue teasing apart the effects of nicotine versus other e-cigarette constituents, mice were exposed to either e-cigarette aerosol containing nicotine (E-cig) or without nicotine (Vehicle).

## Methods

### Murine E-cigarette Exposures

C57BL/6 6–8 week-old male mice (Harlan) were exposed to e-cigarette aerosols or air for 60 minutes daily, 5 days/week, for 12 weeks using the whole-body SCIREQ InExpose System (Emka). Prior to starting exposures, mice were acclimated to the whole-body exposure chambers for 30 min daily for 2 days. Nicotine containing e-liquid consisted of 70% propylene glycol (PG), 30% glycerin (Gly), and 6 mg/ml base nicotine (Sigma). Vehicle mice were exposed with 70:30 PG:VG e-liquid without nicotine, while control mice were exposed to environmental air alone. Aerosols were generated with an e-cigarette Box Mod device with a standard tank (1.8 Ω) and AC power. Pneumatic pressure and puff topography was modeled on human e-cigarette vapers and mirrored our previous studies^18,24^. In brief, e-cigarettes were activated 3 times per minute for 4 seconds at 2 L/min, followed by room air for 16 seconds. Mice were placed in prewarmed cages for 30 min after exposures. After the final exposure, mice were euthanized and kidney and liver tissues were harvested with half placed into 4% paraformaldehyde at 4°C and half snap frozen and stored at −80°C. Fixed tissues were transferred into PBS at 24 hours, paraffin embedded, sliced and stained with H&E and Masson’s Trichrome. Frozen tissue underwent total RNA isolation (Qiagen) followed by bulk RNAseq (Illumina NovaSeq X Plus). All animal experiments were conducted in accordance with the National Institutes of Health *Guide for the Care and Use of Laboratory Animals* under protocols approved by the Institutional Animal Care and Use Committee at the University of California San Diego.

### Quantification of Structural and Fibrotic Changes

Quantification analysis of collagen in Masson Trichrome stained tissue was done using QuPath software. First, the calculate intensity feature was used to quantify the intensity of blue stain present in each organ tissue section. The calculate intensity tool identified and outlined/annotated all detectable cells in each microscopy image and calculated the intensity of blue stain present yielding mean gray value (ratio of pixel area of the stain of interest to the total pixel area of the image). Mean gray values to assess for collagen content differences in kidneys and livers between Air (control), Vehicle and E-cig (vehicle + nicotine) exposures were analyzed via non-parametric Kruskal-Wallis test (GraphPad Prism). There were no structural differences in H&E stained liver and kidney tissue from Air, VEH and E-cig exposed mice and no differences in levels of collagen within Trichrome stained liver and kidney tissue from Air, VEH and E-cig exposed mice (data not shown).

### RNA Extraction

Liver and kidney samples were weighed, sectioned into 2–4 mm^3^ pieces, 600 microliters Trizol added and tissue homogenizer applied. The homogenizer was cleaned with RNAse away, 70% ethanol, RNAse away, and RNAse/DNAse free water in-between samples. Ethanol (600 uL) was added and samples centrifuged at 15,000 rpm for 1 minute. Samples were transferred to Zymo Spin columns, 80 uL of DNAse I Reaction Mix (Qiagen) added and samples incubated at room temperature for 15–20 minutes. RNA Prep Buffer (400uL) was added and samples centrifuged for 1 minute. RNA Wash Buffer (700uL) was added to each column and samples were centrifuged for 1 minute. After another RNA Wash Buffer (400uL) application, samples were centrifuged for 2 minutes. Column filters were transferred to new RNAse free tubes and centrifuged dry for 5 minutes. DNAse/RNAse free water (50uL) was added to each column filter, incubated at room temperature for 2 minutes, and centrifuged for 3 minutes. The flowthrough was re-eluted 1 time and the concentration of RNA was measured (Nanodrop), prior to submission of samples to the UC San Diego IGM Core for transcriptomics (1.25B reads per lane, Illumina NovaSeq X Plus).

### RNA Sequencing Analysis

RNA sequencing libraries were prepared using 50-base-pair single-end reads with an average sequencing depth of approximately 50 million reads per sample. The raw sequencing reads were aligned to the mouse reference genome (GRCm38) using the Bowtie version 1.3.0 aligner^30,31^ and quantified with RSEM version 1.3.0^32^. Gene annotations were derived from a comprehensive mouse gene annotation dataset provided by the GENCODE project^33^. This dataset includes detailed information about known protein-coding genes, non-coding genes, and their transcript variants in the mouse genome, enabling accurate mapping of sequencing reads to specific genomic features. To reduce noise in the dataset, only genes with counts per million (CPM) values greater than 100 in at least two samples were retained. This filtering ensured that only genes with sufficient expression levels across samples were included in the analysis. Normalization of gene expression data was performed to account for differences in library size across samples, which could arise due to varying sequencing depths or RNA quality. This process involved scaling the raw counts for each sample to make them comparable by calculating size factors. These size factors are determined by estimating the median ratio of observed gene counts in each sample relative to a reference sample, effectively adjusting for sample-to-sample variability while preserving biological differences. Differential gene expression (DGE) analysis was then conducted using exact tests for pairwise comparisons between experimental groups. Genes were considered significantly differentially expressed if their adjusted p-values, corrected using the false discovery rate (FDR), were less than or equal to 0.05, and their absolute log-fold change was greater than or equal to 1.

### A priori gene set analysis

To enhance statistical power, a separate analysis was conducted on a curated a priori gene set specific to liver and kidney fibrosis, sourced from published literature and created prior to the generation of transcriptomic data. DGE tests were conducted exclusively within these gene sets, allowing more precise detection of significant changes in pathways of interest. Overlapping genes with significant differential expression across multiple comparisons were identified and visualized.

### Biological pathway analysis

Following differential gene expression analysis, pathway enrichment analysis was performed to contextualize systematic alterations within established biological processes. All genes with nominal significance (p < 0.05) in each comparison (E-cig vs Air and VEH vs Air for both kidney and liver tissues) were included as input. Analyses were conducted using the pathfindR R package^34^, which systematically identifies active subnetworks and enriched pathways based on the Kyoto Encyclopedia of Genes and Genomes (KEGG) database^35^. Pathways with adjusted p-values < 0.05 were considered significantly enriched. To reduce redundancy and improve interpretability, functionally similar pathways were clustered based on gene overlap, and representative pathways from each cluster were retained for reporting.

### Data visualization

All analyses and visualizations were conducted using R v4.2.1. Principal component analysis (PCA) was performed on normalized gene expression data to evaluate clustering patterns across experimental groups. Volcano plots were generated to display −log10 transformed p-values against LogFC values of the a priori gene set, with significant genes highlighted. Scatter plots were made to depict associations between fold changes of gene expression in liver and kidney tissues.

## Results

### Kidney and Liver Transcriptomic Profiles

**Figure 2.**
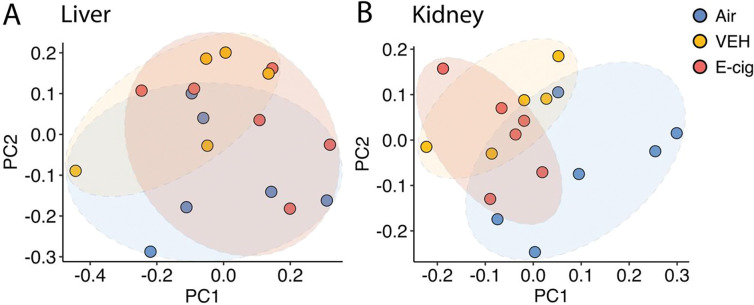
Principal Component Analysis (PCA) of gene expression profiles in liver and kidney tissues in the setting of chronic exposure to e-cigarette vapor. **A.** PCA plot of gene expression data from liver tissue. Each point represents a sample, and the clustering reflects variation in the overall transcriptomic profile across the three experimental conditions: Air-exposed (blue), vehicle control (VEH, yellow), and e-cigarette vapor-exposed (E-cig, red). Principal Component 1 (PC1) and Principal Component 2 (PC2) account for most of the variance in gene expression among samples. Clustering patterns highlight the similar transcriptomic profiles across groups. **B.** PCA plot of gene expression data from kidney tissues. The separation along PC1 and PC2 indicates condition-specific differences in transcriptomic profiles, with the E-cig and VEH groups having some separation in clustering away from the Air group. n = 5–6 per group.

Principal component analysis (PCA) plots highlight global transcriptomic patterns across exposure conditions in liver and kidney tissues. Global gene expression in liver tissue from E-cig exposed mice was similar to that from Air and VEH (no nicotine) groups, indicating a lack of broad transcriptomic changes driven by nicotine or other e-cigarette vapor chemicals (Figure 2A). In the kidney, tissue gene transcription in E-cig and VEH exposed mice predominantly clustered separately from Air, indicating transcriptomic changes driven by inhaled chemicals with e-cigarette vapor (Figure 2B). The overlap between E-cig and VEH gene expression patterns demonstrates that non-nicotine chemicals may be the primary drivers in kidney tissue (Figure 2B).

**Figure 3:**
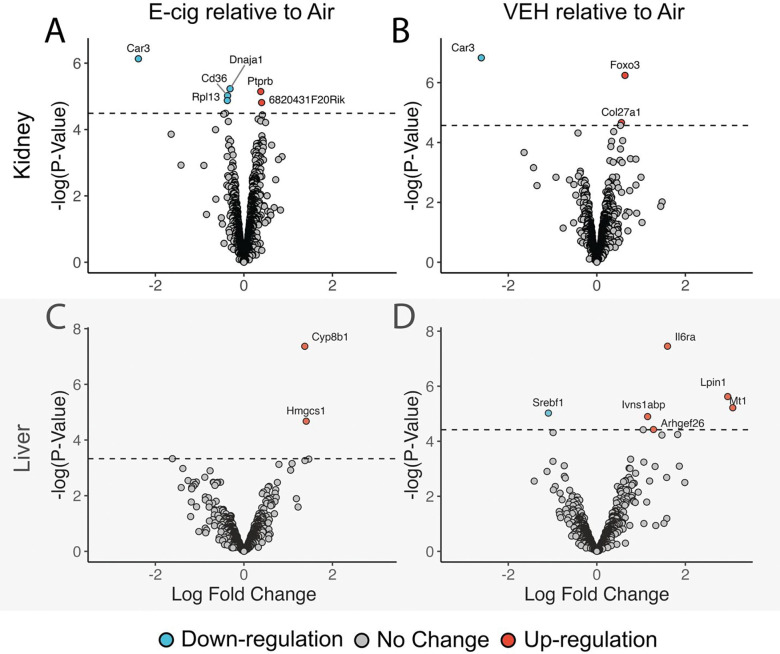
Differential gene expression in liver and kidneys of mice exposed chronically to e-cigarette vapor. Volcano plots showing the results of differential gene expression (DGE) analyses in kidney (upper panels **A and B**) and liver (bottom panels **C and D**) tissues when comparing e-cigarette vapor (E-cig, left panels **A and C**) and vehicle (VEH, right panels **B and D**) exposures, all analyzed relative to tissues from air control mice. Each point represents an individual gene, with the x-axis indicating the log fold change (LogFC) and the y-axis showing the −log10 transformed p-value. Genes with adjusted p-values (FDR) ≤ 0.05 and absolute LogFC ≥ 1 are considered significantly differentially expressed. The dashed horizontal line represents the significance threshold at −log10(p-value) corresponding to FDR = 0.05. This significance threshold was established using a false discovery rate (FDR). The FDR differs for each comparison, based on the distribution of the statistics, such that it can be ensured that genes above the FDR threshold have p-values that are truly significant after the FDR for their own comparison is used for the correction. Genes are classified as significantly down-regulated (blue), significantly up-regulated (red), or not significantly differentially expressed (grey). These plots highlight distinct transcriptomic changes induced by E-cig and VEH exposures, with differences observed between tissues (**A&B** versus **C&D**) and exposure types (**A&C** versus **B&D**). Upregulated and downregulated genes are more prominent under E-cig exposure (**A&C**; containing nicotine), indicating a stronger transcriptomic response compared to VEH (**B&D**; no nicotine).

Chronic E-cig exposure resulted in the differential expression of 10 genes in kidney tissue (Figure 3A), whereas VEH (no nicotine) exposure changed expression of three genes (Figure 3B). One gene was downregulated by both E-cig and VEH exposure, Car3, demonstrating that non-nicotine components of e-cigarette vapor contributed to this change. Other gene expression changes included upregulation of genes involved in extracellular matrix organization, renal fibrosis, and immune regulation, suggesting that chronic e-cigarette exposure induces molecular pathways contributing to kidney fibrosis and inflammation (Table 1).

In the liver, chronic E-cig exposure induced upregulation of two genes, Cyp8b1 and Hmgcs1 (Figure 3C), while VEH exposure altered seven (Figure 3D). Many of these genes were linked to metabolic regulation, lipid homeostasis, and oxidative stress pathways, highlighting a unique tissue-specific response to chronic e-cigarette exposure (Table 1).

Prior to obtaining transcriptomic data, a total of 335 genes were pre-curated in the a priori gene list, including subsets associated with liver fibrosis, kidney fibrosis, and shared biological processes between liver and kidney tissues (Supplementary Table 1). After applying the false positive corrections based on the genes included in the apriori list, none of the overlapping genes came out as significant. Notably, *Col4a1* showed nominal significance of overexpression (p = 0.040; LogFC = 0.177) after E-cig exposure in the kidney, suggesting potential involvement in fibrotic processes. Given its established role as a structural component of basement membranes, this modest increase may reflect early extracellular matrix remodeling and potential involvement in fibrotic processes^36^.

### Pathway enrichment analysis

**Figure 4:**
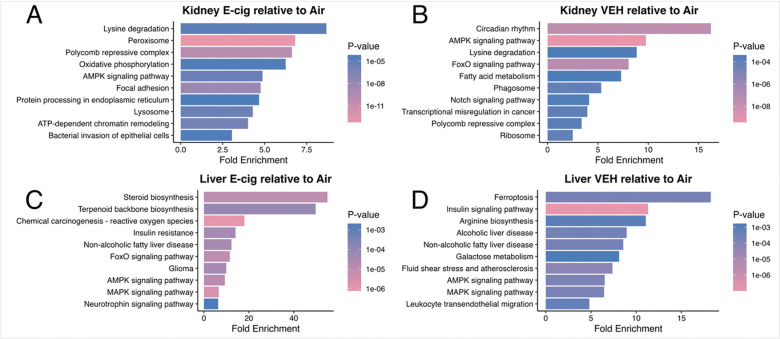
Pathway enrichment analysis of kidney and liver transcriptomes changes following chronic e-cigarette exposure. Top 10 KEGG pathway enrichment results based on fold enrichment are shown for (A) kidney E-cig vs. Air, (B) kidney VEH vs. Air, (C) liver E-cig vs. Air, and (D) liver VEH vs. Air. Bar length indicates fold enrichment, and color denotes p-value significance. E-cig exposures in the kidney were associated with enrichment of pathways related to oxidative stress, mitochondrial function, and extracellular matrix remodeling, while VEH exposures primarily affected circadian, metabolic, and signaling pathways. In the liver, E-cig exposures enriched lipid biosynthesis, insulin resistance, and ROS-related pathways, whereas VEH exposures enriched ferroptosis, insulin signaling, and fatty liver disease pathways.

Pathway analysis further contextualized the transcriptomic changes induced by chronic e-cigarette vapor exposure. In the kidney, E-cig exposure enriched pathways including lysine degradation, peroxisome, oxidative phosphorylation, AMPK signaling, focal adhesion, and protein processing in the endoplasmic reticulum (Figure 4A). In contrast, VEH exposure significantly enriched pathways related to circadian rhythm, AMPK signaling, lysine degradation, FoxO signaling, and fatty acid metabolism, indicating disrupted energy homeostasis and transcriptional control of lipid processing (Figure 4B). These signatures point to broad perturbations in mitochondrial function, oxidative stress, and extracellular matrix interactions, suggesting that nicotine-containing aerosols amplify both metabolic and structural remodeling processes in renal tissue. In the liver, E-cig exposure produced enrichment in steroid and terpenoid backbone biosynthesis, chemical carcinogenesis (reactive oxygen species), insulin resistance, and non-alcoholic fatty liver disease (NAFLD), alongside signaling pathways including FoxO, AMPK, and MAPK (Figure 4C). These findings suggest that nicotine-containing aerosols exacerbate metabolic dysfunction and oxidative stress, particularly through mitochondrial and ROS-driven pathways, while also disturbing cholesterol and lipid biosynthetic processes. In contrast, VEH exposure enriched pathways such as ferroptosis, insulin signaling, arginine biosynthesis, alcoholic liver disease, and NAFLD (Figure 4D). These results highlight altered iron-dependent oxidative stress, impaired glucose and lipid metabolism, and susceptibility to hepatic injury even in the absence of nicotine. Together, these results demonstrate that both nicotine and non-nicotine e-cigarette vapor constituents disrupt central metabolic, oxidative, and stress-response pathways in kidney and liver tissues, with partially overlapping but distinct mechanistic signatures. All enriched biological pathways can be found in Supplementary Table 4.

### Divergent gene expression between liver and kidney tissues

**Figure 5:**
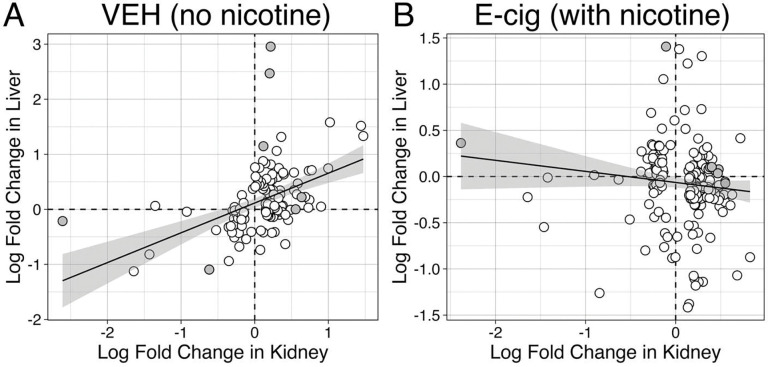
Correlation of DGE between liver and kidney tissues in the setting of e-cigarette vapor inhalation with and without nicotine. Scatter plots comparing the log fold change (LogFC) of genes differentially expressed in liver and kidney tissues for vehicle control (VEH) and e-cigarette vapor (E-cig) exposure conditions. Linear regression was performed to assess the relationship between gene expression changes in liver and kidney tissues. Each point represents a gene with its LogFC in the kidney plotted on the x-axis and its LogFC in the liver plotted on the y-axis. The dashed horizontal and vertical lines indicate LogFC = 0, representing no change in gene expression. A linear regression line (solid black) is overlaid with a shaded 95% confidence interval. **A.** In VEH exposed mice, regression analysis identified a strong positive correlation between LogFC in kidney and liver tissues (slope = 0.541, p < 0.001, R^2^ = 0.217), indicating that genes tend to exhibit coordinated expression changes across the two tissues in the setting of chronic exposure to inhaled PG:VG without nicotine (VEH). **B.** Regression analysis revealed a weaker and statistically non-significant correlation between LogFC in kidney and liver tissues (slope = −0.120, p = 0.103, R^2^ = 0.012) of mice chronically exposed to nicotine containing e-cigarette aerosols (E-cig). This suggests more divergent gene expression patterns between liver and kidney tissues in the setting of chronic nicotine containing E-cig exposure.

Scatter plots of log fold change (LogFC) values between liver and kidney tissues (Figure 5) highlight divergent transcriptional responses across tissues and exposure conditions. With chronic no-nicotine VEH exposure (Figure 5A), a significant positive correlation (slope = 0.541, R^2^ = 0.217, p < 0.001) was observed, indicating comparable transcriptional responses in liver and kidney tissues. In contrast, in the setting of chronic exposure to nicotine containing EV (Figure 5B), there was no correlation between liver and kidney (slope = −0.120, R^2^ = 0.012, p = 0.103). These findings suggest that chronic inhalation of nicotine induces tissue-specific gene expression in the liver and kidneys.

## Discussion

The transcriptomic profile changes revealed in this study demonstrate that chronic e-cigarette exposure leads to divergent gene expression changes in liver and kidney tissues, reflecting organ-specific responses to inhaled aerosols.

In terms of the gene-level changes in the kidney, chronic e-cigarette exposure led to molecular changes highlighting the susceptibility of the renal system to fibrotic remodeling and dysfunction. *Carbonic Anhydrase 3* (*Car3*) was significantly downregulated in both E-cig and VEH groups. Because *Car3* plays a crucial role in maintaining acid-base balance and pH homeostasis^37^, its suppression underscores disruption in renal buffering capacity, increasing the risk of metabolic disturbances. Similarly, downregulation of *CD36*, a key regulator of fatty acid metabolism and inflammation, suggests impaired lipid processing and heightened inflammatory responses, both of which contribute to fibrosis^38^.

Reduced expression of *Heat Shock Protein Family Member DNAJB1* (*Dnaja1*), which assists in protein folding and stress response, indicates a diminished ability of kidney cells to cope with oxidative stress and misfolded protein accumulation, processes often implicated in tissue injury and fibrotic progression^39^. Conversely, genes such as *Protein Tyrosine Phosphatase, Receptor Type B* (*Ptprb*) and *Forkhead Box O3* (*Foxo3*) were upregulated, reflecting potential compensatory mechanisms^40^. *Ptprb* upregulation may enhance signaling pathways related to cellular proliferation^40^ while *Foxo3* could support oxidative stress resistance, potentially counteracting some of the adverse effects of e-cig exposure^41^.

Notably, the upregulation of *Collagen Type XXVII Alpha 1 Chain* (*Col27a1*), a major extracellular matrix component, aligns with fibrotic remodeling observed in the kidney^42^. Increased *Col27a1* expression suggests enhanced collagen deposition and extracellular matrix restructuring, hallmark features of fibrosis. These findings collectively emphasize that chronic e-cig exposure alters key pathways in the kidney, promoting inflammation, oxidative stress, and extracellular matrix remodeling, which are central to the development of fibrosis.

In terms of the gene-level changes in the liver, gene expression in liver tissues of mice exposed chronically to e-cigarette aerosols demonstrated changes particularly in pathways associated with lipid metabolism, inflammation, and oxidative stress. *Cytochrome P450 Family 8 Subfamily B Member 1* (*Cyp8b1*), upregulated in EV-exposed mice, plays a pivotal role in bile acid synthesis and cholesterol metabolism^43,44^. Elevated *Cyp8b1* expression may disrupt bile acid homeostasis^43,44^, leading to hepatocyte injury and chronic inflammation, both of which are significant drivers of liver fibrosis. Another critical gene, *3-Hydroxy-3-Methylglutaryl-CoA Synthase 1* (*Hmgcs1*), essential for cholesterol biosynthesis, was also upregulated in EV-exposed mice^45^. Excessive cholesterol synthesis can destabilize cell membranes^45^, triggering the release of inflammatory cytokines and damage-associated molecular patterns (DAMPs). These molecules activate immune responses, amplifying hepatocyte injury and fibrotic progression. Further, elevation of *Interleukin 6 Receptor Subunit Alpha* (*Il6ra*) in livers of VEH mice underscores the role of inflammatory pathways in e-cig-induced liver damage^46,47^. Dysregulated *IL-6/STAT3* signaling has been implicated in various inflammatory and autoimmune conditions, linking e-cig exposure to chronic liver inflammation. Upregulated genes such as *Lipin 1* (*Lpin1*) and *Metallothionein 1* (*Mt1*) point to a complex interplay between lipid metabolism, oxidative stress, and inflammation^48–52^. While *Lpin1* supports lipid biosynthesis and fatty acid oxidation, its dysregulation can exacerbate metabolic imbalances. Mt1, a key regulator of metal homeostasis and oxidative stress response, suggests an adaptive attempt to mitigate cellular damage induced by chronic e-cig exposure. Conversely, the downregulation of Sterol Regulatory *Element-Binding Transcription Factor 1* (*Srebf1*), which regulates lipid homeostasis, reflects impaired lipid production, potentially contributing to metabolic disorders such as non-alcoholic fatty liver disease (NAFLD)^53,54^. These findings underscore the multifaceted impact of e-cig exposure on the liver, revealing disruptions in lipid metabolism, inflammation, and oxidative stress pathways, all of which contribute to fibrotic disease progression.

Pathway enrichment analyses highlighted that in the kidney, non-nicotine vehicle exposures preferentially disrupted circadian rhythm, AMPK signaling, lysine degradation, FoxO signaling, and fatty acid metabolism, pathways central to renal energy balance and nutrient sensing. This suggests that even nicotine-free e-cigarette aerosols can disrupt metabolic timing, stress adaptation, and lipid handling in the kidney, predisposing to long-term renal dysfunction^26,55^. In contrast, nicotine-containing exposures perturbed lysine degradation^56,57^, peroxisome activity, oxidative phosphorylation, focal adhesion, and protein processing in the endoplasmic reticulum, implicating nicotine in amplifying oxidative stress^58^, mitochondrial injury, and extracellular matrix remodeling, processes that contribute to kidney fibrosis and loss of function. In the liver, vehicle exposures strongly enriched pathways including ferroptosis^59^, insulin signaling, arginine biosynthesis, alcoholic liver disease, and NAFLD. These findings indicate that the solvents and additives in e-cigarette aerosols, independent of nicotine, can promote iron-dependent oxidative injury, disrupt amino acid and glucose metabolism, and drive disease pathways resembling both alcoholic and non-alcoholic fatty liver injury. By comparison, nicotine-containing exposures targeted steroid and terpenoid backbone biosynthesis, chemical carcinogenesis (reactive oxygen species), insulin resistance, NAFLD, and MAPK signaling. This pattern suggests that nicotine amplifies hepatic metabolic stress by perturbing cholesterol and lipid biosynthesis, enhancing ROS-mediated carcinogenic signaling, and promoting insulin resistance, all of which can accelerate progression to steatosis, inflammation, and fibrosis. These findings emphasize the systemic and organ-specific risks of e-cigarette use, reinforcing the need for further research into its long-term health impacts^60^.

Even though we minimized the time between euthanasia and organ harvest, it is possible that the time between euthanasia, harvest, and placement into liquid nitrogen may have compromised RNA integrity or led to ischemia and hypoxia related gene expression changes. Another limitation is that these data capture RNA expression at a single time point (3 months of e-cigarette aerosol inhalation), offering only a snapshot of the molecular changes occurring due to vaping. In the future, a longitudinal study would enable characterization of dynamic changes in inflammatory and fibrotic pathways over time, providing a more comprehensive understanding of e-cig-induced damage. For example, if e-cigarette aerosol inhalation induced inflammation in the acute (1–3 days) or sub-acute (1–2 weeks) phase, our data would only capture RNA expression post-inflammation resolution, thus underestimating or missing inflammatory patterns. Additionally, we did not evaluate for a dose response of nicotine in e-cig aerosols. Given nicotine’s known biological effects, future studies should explore how varying concentrations influence gene expression and fibrosis-related pathways. Despite these limitations, this study provides critical insights into the molecular effects of chronic e-cig exposure, particularly its role in promoting fibrosis in the liver and kidneys and enhancing our understanding of the systemic impact of e-cigarettes.

## Conclusions

Just as cigarette smoking impacts cells and tissues across the body, e-cigarette vaping appears to also have broad systemic effects. While defining cellular and molecular effects of e-cigarette vaping on the lungs is critical, as this organ system has the highest level of exposure to e-cigarette chemicals, our work demonstrates the need to look at organs across the body, to ensure that we best understand the potential long-term effects of vaping. With these data in hand, we can better inform the public and guide public policy.

## Supplementary Material

Supplementary Files

This is a list of supplementary les associated with this preprint. Click to download.

• KeyMessagesEcigFibrosis8272025.docx

• SupplementalTable1.pdf

• SupplementalTable2.pdf

• SupplementalTable3.pdf

• SupplementalTable4.pdf

**Supplemental Table 1:** Kidney Gene List for A priori Gene Set Analysis

**Supplemental Table 2:** Liver Gene List for A priori Gene Set Analysis

**Supplemental Table 3:** Common Genes from Final Kidney and Liver Gene Lists Used for A priori Gene Set Analysis

**Supplementary Table 4:** All Biological Pathways Enriched in the Kidney and Liver

## Figures and Tables

**Table 1.** Summary of significantly differently, regulated genes by organ and treatment group. Changes associated with e-cigarette vapor containing nicotine (EV) are indicated in bold blue, while those associated with vehicle (VEH) are in black font.

Organ	Treatment	Gene Name	Symbol	LogFC	P-Value	FDR
Kidney	**E-cig (with nicotine)**	**ENSMUSG00000027559**	**Car3**	**−2.38**	**7.37E-07**	**1.46E-03**
		**ENSMUSG00000102307**	**Gm38194**	**0.51**	**8.86E-07**	**1.75E-03**
		**ENSMUSG00000113673**	**Gm47486**	**0.55**	**1.18E-06**	**2.33E-03**
		**ENSMUSG00000028410**	**Dnaja1**	**−0.31**	**5.91E-06**	**1.17E-02**
		**ENSMUSG00000020154**	**Ptprb**	**0.38**	**7.17E-06**	**1.41E-02**
		**ENSMUSG00000111481**	**Gm47652**	**0.48**	**9.33E-06**	**1.84E-02**
		**ENSMUSG00000002944**	**Cd36**	**−0.37**	**9.56E-06**	**1.89E-02**
		**ENSMUSG00000000740**	**Rpl13**	**−0.37**	**1.34E-05**	**2.64E-02**
		**ENSMUSG00000071796**	**6820431F20Rik**	**0.4**	**1.54E-05**	**3.03E-02**
		**ENSMUSG00000110779**	**Gm48271**	**0.47**	**1.85E-05**	**3.64E-02**
	Vehicle (VEH)	ENSMUSG00000027559	Car3	−2.61	1.48E-07	2.92E-04
		ENSMUSG00000048756	Foxo3	0.64	5.72E-07	1.13E-03
		ENSMUSG00000045672	Col27a1	0.56	2.16E-05	4.28E-02
Liver	**E-cig (with nicotine)**	**ENSMUSG00000050445**	**Cyp8b1**	**1.37**	**4.30E-08**	**5.71E-05**
		**ENSMUSG00000093930**	**Hmgcs1**	**1.41**	**2.11E-05**	**2.80E-02**
	Vehicle (VEH)	ENSMUSG00000027947	Il6ra	1.6	3.52E-08	4.67E-05
		ENSMUSG00000105703	Gm43305	2.47	2.06E-07	2.74E-04
		ENSMUSG00000020593	Lpin1	2.96	2.38E-06	3.15E-03
		ENSMUSG00000031765	Mt1	3.07	6.05E-06	8.02E-03
		ENSMUSG00000020538	Srebf1	−1.09	9.45E-06	1.25E-02
		ENSMUSG00000023150	Ivns1abp	1.15	1.27E-05	1.67E-02
		ENSMUSG00000036885	Arhgef26	1.28	3.74E-05	4.94E-02

## Data Availability

Data will be uploaded to a publicly available data repository upon publication. Authors will also share the data upon reasonable request.
